# Opportunities and challenges of global health diplomacy for prevention and control of noncommunicable diseases: a systematic review

**DOI:** 10.1186/s12913-021-07240-3

**Published:** 2021-11-04

**Authors:** Mohsen Asadi-Lari, Ahmad Ahmadi Teymourlouy, Mohammadreza Maleki, Mahnaz Afshari

**Affiliations:** 1 Department of Epidemiology, School of Public Health, Iran University of Medical Sciences, Tehran, Iran; 2 Department of Health Service Management, School of Health Management and Information Sciences, Iran University of Medical Sciences, Tehran, Iran

**Keywords:** Systematic review, Global health diplomacy, Noncommunicable diseases

## Abstract

**Background and aim:**

The growing globalization has changed the goals and methods of diplomacy. Due to the challenges and complexities of dealing with noncommunicable diseases (NCDs) at the national and international levels, policy makers require global health diplomacy (GHD) to achieve the goals of prevention and control of NCDs. The purpose of this systematic review was to identify the challenges and opportunities in GHD for NCDs.

**Methods:**

A systematic review of articles was conducted by searching MEDLINE via PubMed, Web of Science, Scopus, and Embase and by using Google and Google Scholar search engines. Additional articles were identified by reviewing reference lists and a number of special journals. The inclusion criteria include literature published in English from 2007 to 2020, and the exclusion criteria are literature published in any language other than English, absence of full text, dissertations, and duplicates. Overall, 32 articles met the requirements for inclusion in this review and were analyzed using content analysis in MAXQDA 10.

**Findings:**

There are 32 published articles on GHD for NCDs. Identified challenges were classified into three levels: global (global health governance), national (Governance at the state level, health sector, and civil society), and industry. The progress on global health issues has created opportunities for the development of GHD for the prevention and control of NCDs. These opportunities were divided into three levels: international, national, and individual level.

**Conclusion:**

Various challenges at the global level, national level, and industry led to less engagement of policymakers in GHD for prevention and control of NCDs and, as a consequence, a less coordinated approach to address prevention and control of NCDs worldwide. So, there is a need for more efforts of collective action and negotiation for tackling NCDs. Policymakers and managers of the health system should increase the advocacy, building a coalition with civil society, use negotiation and diplomacy to engage with other sectors and organizations, manage industry conflicts, and leverage foreign policy to promote health and welfare.

**Supplementary Information:**

The online version contains supplementary material available at 10.1186/s12913-021-07240-3.

## Introduction

Noncommunicable diseases (NCDs) are a major cause of death and disability worldwide. In developing countries, the burden of NCDs is increasing rapidly and will have significant social, economic, and health consequences [1]. For instance there has been an increase in the burden of NCDs in countries of Sub-Saharan Africa during the last decades, pushed through growing prevalence of cardiovascular threat factors which includes unhealthy diets, decreased physical activity, hypertension, obesity, diabetes, dyslipidaemia, and air pollution [2, 3]. As another example, the rising trend of deaths and years of disabilitydue to NCDs adjusted over the last decades is an impressive hazard to Iran. According to the Global Burden of Disease study, the mortality rate from non-communicable diseases has steadily increased from 50% in 1990 to 82% in 2017 in Iran [4]. NCDs not only lead to premature death but also cause significant disability [5]. Due to the sharp increase in mortality rates due to NCDs over the past two decades, especially in developing countries, the World Health Organization (WHO) has declared NCDs a top health priority for every country [6]. Cardiovascular diseases, cancer, chronic respiratory diseases, and diabetes are four major NCDs [7]. Primary prevention is the key to controlling the global epidemics of NCDs. The main goal is to avert and control these epidemics wherever possible, and surveillance is crucial to its success [8]. The increasing prevalence of NCDs, especially in recent decades, has raised concerns about the health systems around the world and those involved with health policy making. The United Nations (UN) and WHO are among the bodies that have developed programs to prevent and control NCDs worldwide and have been working to promote health in this area [9, 10]. Evidence of this is the holding of high level meetings and the development of guidelines that target NCDs.

WHO has identified several health priority areas in cooperation and consultation with its member states. Prevention and control of NCDs is one of these priority areas that have been placed on the global health agenda and health diplomacy [10]. Tackling NCDs requires intersectoral collaboration. The prioritization and emphasis on a global commitment to tackle NCDs has led to the development of effective strategies such as a multi-stakeholder structure to serve as a holistic platform that enables transparency and accountability in negotiating policy space for NCDs [11]. One instance of successful global health negotiation and diplomacy is the Port of Spain Declaration on NCDs, which was elevated to a global level and led to the 2011 High Level Meeting on the Prevention and Control of Non-communicable Diseases by the UN [11, 12]. Therefore, global health diplomacy (GHD) is crucial to ensuring political commitment and intersectoral collaboration to tackle NCDs on a global scale [12].

There is emerging evidence that global trade is associated with the rise of chronic diseases in many low and middle-income countries (LMICs) [13]. In the short or medium term,the liberalization of food trade in 15 developing countries including Chile, Guatemala, Guyana, Peru, Cameroon, Ghana, Kenya, Malawi, Morocco, Nigeria, Senegal, Tanzania, Uganda, China, and India Reform can cause damage to food safety, if it’s far brought without a coverage package deal designed to offset the negative consequences of liberalization [14–16]. In addition, supporting evidence from India, the Pacific Islands, and Colombia shows that the growth of international trade has shifted the diet from a healthy local diet to a high-fat diet [17]. This is in part due to the global prevalence of unhealthy lifestyles and health-damaging products and is particularly challenging for countries that are vulnerable to the burdens of communicable diseases. To reduce the burden of NCDs, it is essential for health policy makers to use negotiation and diplomacy to interact with trade policy makers regarding the health effects of international trade agreements and ensure the flow of health-promoting products. Prevention and treatment of chronic diseases are currently on global policy agendas, as highlighted in the 2011 UN Summit on NCDs. There is a need for a more coordinated approach to regulating trade-related risk factors and increasing engagement between health and trade policy sectors within and between nations [13].

Overall, it can be concluded that the discussion of NCDs is not limited to one country or region and is a problem that all countries around the world are struggling with. Reducing the impact of NCDs on individuals and communities requires a comprehensive approach such as GHD that engages all sectors, including health, finance, foreign affairs, education, agriculture, planning and others to reduce the risks associated with NCDs and promote disease prevention and control interventions. Otherwise, it will be impossible to achieve the objectives set by WHO for the prevention and control of NCDs.

The UN Political Declaration was an important step in recognition of NCDs as a global health challenge in the twenty-first century that has undermined social and economic development and has threatened the achievement of development goals. On the other hand, the link between NCDs and Sustainable Development Goals (SDGs) as well as the unfinished agenda of Millennium Development Goals is another reason for the placement of NCDs on the global agenda and the emphasis on controlling them with collaboration and appropriate policies at the national and international levels [18].

The Political Declaration highlights strengthening health systems towards the provision of equitable, universal health coverage and promoting affordable access to prevention, treatment, care and support related to NCDs, especially cancer, cardiovascular diseases, chronic respiratory diseases, and diabetes, as one of the main challenges to sustainable development in the twenty-first century. It also commits to establishing or strengthening national and international multisectoral policies for the prevention and control of NCDs [9]. NCDs are a threat to the UN Millennium Development Goals and post-2015 development plans. Using high-impact, essential interventions such as GHD for NCDs to prevent and control these diseases is an excellent economic investment because they can reduce the need for more expensive treatments if provided early to patients.

One of the challenges in tackling NCDs is the complexity of these diseases as they have a wide range of risk factors from global (e.g., multinational fast food companies) to local (unwalkable streets) levels. In addition, not all chronic diseases can be prevented. Due to these complexities, it is difficult to specify the goals and required funds for the prevention and control of NCDs. The global response to these diseases must focus on generating multisectoral evidence regarding the transnational factors that contribute to the rise in NCDs as well as the potential policies proposed for controlling them [19]. Development and adoption of a global reaction to the upward push of chronic diseases in industrialized and developing countries calls for policy makers to engage in GHD. Successful developments in GHD depend on the performance of and respectful relationships among the stakeholders, and global health diplomats must have a deep understanding of the institutional structures and the ways in which these relationships work [12, 20]. The purpose of this systematic review is to identify the challenges and opportunities in GHD for NCDs.

## Methods

The present research was a systematic review of the literature for identiffing opportunities and challenges of global health diplomacy (GHD) for the prevention and control of noncommunicable diseases (NCDs), including cardiovascular diseases, cancer, respiratory diseases and cancer as well as their risk factors, including tobacco use, excessive salt and fat intake, alcohol abuse, and physical inactivity. This review covered the period 2007–2020.

The PICO framework (patient/population/participant problem, intervention, comparison, outcome) will be applied to the formulation of questions and will facilitate the clarification of search strategies. The PICO frameworkof this study have been given in supplementary file 1. Relevant articles were identified by searching MEDLINE database via PubMed, Web of Science, Scopus, and Embase along with Google and Google Scholar search engines. The keywords used in searches included English MeSH and common terms related to the topic, including “Diplomacy” OR “Internationality” OR “foreign policy” OR “foreign affairs” OR “international relations” OR “international politics” OR “statesmanship” OR “statecraft” OR “Health Diplomacy” OR “Medical Diplomacy” OR “Negotiations” OR “Multilateral Engagement” OR “Bilateral Agreements” and “Drinking Behavior” OR “Alcoholic Beverages” OR “Smoking” OR “Smokers” OR “Feeding Behavior” OR “Diet” OR “Obesity” OR “Food” OR “Fast Foods” OR “Sugars” OR “Sodium, Dietary” OR “Exercise” OR “Life Style” OR “Healthy Lifestyle” OR “Sedentary Behavior” OR “Alcohol Drinking” OR “Tobacco Use” OR “Tobacco Products” OR “Tobacco” OR “Noncommunicable Diseases” OR “Non-communicable Diseases”. The reference lists of identified articles were also manually searched to find more relevant papers.

Research studies, commentary, and review articles are all included in the qualitative synthesis. Because early searches of the literature revealed that the majority of relevant research were undertaken after 2007, the inclusion criteria for this study comprised literatures published in English and studies published between 2007 and 2020. The criterion for exclusion were publishing in any language other than English, absence of full text availability, dissertations, and duplicates.

**Fig. 1:**
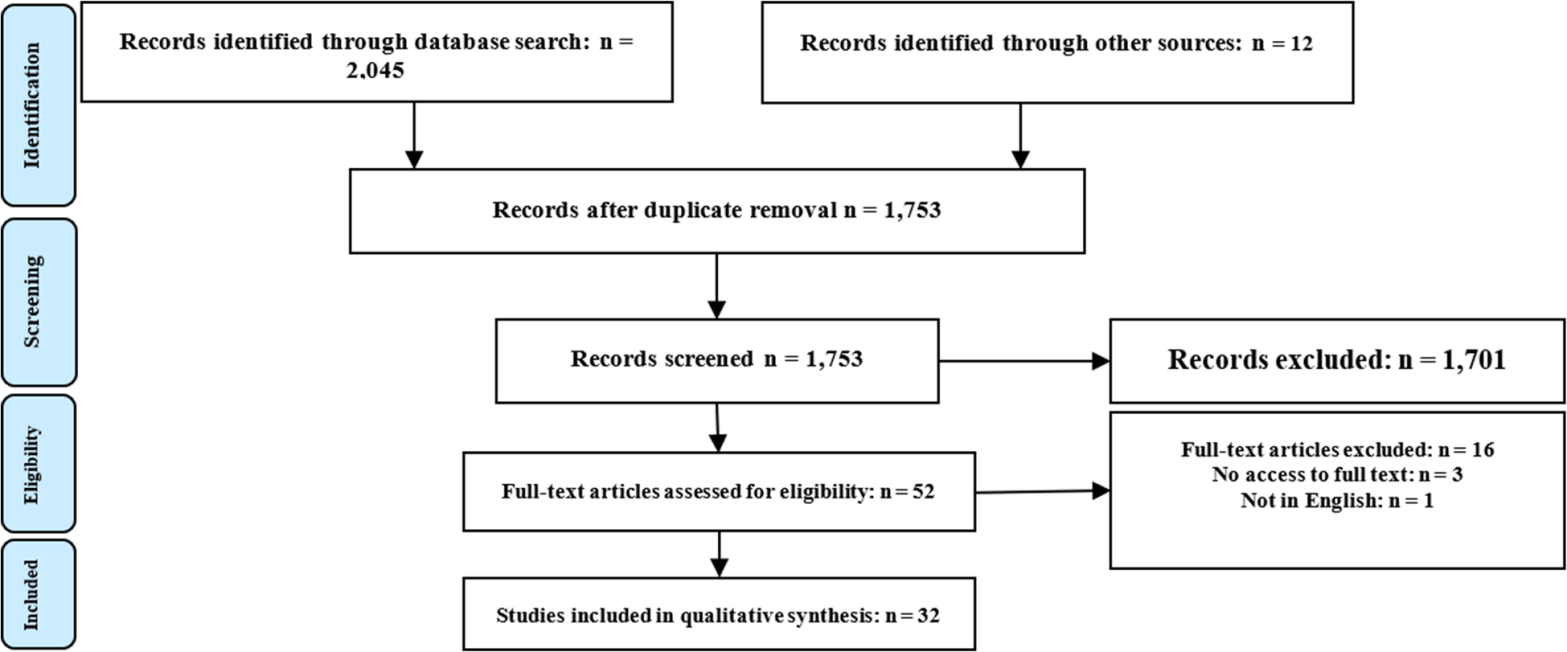
PRISMA Flow Diagram: Database search and article selection process

There have been a total of 2045 items extracted. Based on the inclusion and exclusion criteria, the titles and abstracts of the papers were reviewed, leaving 54 articles for full-text review. 20 articles were eliminated because they were unavailable or duplicates and one article was excluded due to a non-English language (Russian) [21]. Finally, 32 papers were chosen for review. As a PRISMA Flow Diagram, Fig. 1 depicts the database search and article selection process [22].

32 articles have been entered into the quality assessment stage. Quality become assessed independently with the aid of using research the use of the 15-point instrument of Mitton et al. [23]. Criteria for quality assessment included: literature review and identity of studies gaps; research questions, hypotheses, and design; population and sampling; data collection process and instruments; and analysis and reporting of results and every object is given a rating of 0 (not present or reported), 1 (present but low quality), 2 (present and midrange quality), or 3 (present and high quality). Disagreements have been resolved via way of means of discussion and, while essential, by consulting a third evaluation author. Given that the review was qualitative, articles have been not eliminated at this stage, however greater weight was given to articles with a quality score of 10 or above in the data analysis and interpretation of results.

In the next stage, a qualitative content analysis of the articles was performed. The obtained information was coded and analyzed using MAXQDA 10 software for Windows (VERBI GmbH, Berlin, Germany), and the themes and subthemes related to each article were extracted to identify their relationships, patterns, and core meanings. McQueen’s analytical framework for intersectoral governance [24] was used to explain the challenges of global health diplomacy for non-communicable diseases. Opportunities were also classified into three levels: international, national, and individual.

## Results

Between 2007 and 2020, a total of 32 articles have been published on GHD for NCDs. Identified challenges were classified at three levels, including global (global health governance), national (governance at the state level, health sector, and civil society), and industry. McQueen’s analytical framework for intersectoral governance was used to describe the challenges of GHD for NCDs at the global health governance and governance at the state level. This framework consists of governance structures that countries adopt in order to guide multisectoral actions. Identified actions include evidence support, coordination, advocacy, financial support, legal mandates, implementation, monitoring, and evaluation. Tables 1, 2 and 3 present the challenges of GHD for NCDs at different levels along with the identified solutions.

**Table 1:** Challenges of GHD for NCDs at the international level

Themes	Subthemes	Challenges	Solutions
**Global Health Governance**	Evidence Support	▪ Insufficient scientific research ▪ Many countries lack the necessary technical expertise, resources, research capacity, and data required to overcome NCD challenges ▪ GHD negotiations depend upon clinical or epidemiological evidence, and the quality or type of the evidence can limit or expand policy options ▪ Potential differences between national and global priorities ▪ Lack of informations and input from LMICs in stakeholder negotiations	▪ Creating an evidence base through focused primary research that involves all stakeholders and affects the infrastructural challenges to global NCD policy and governance ▪ Combining existing resources and skills in order to negotiate access to the expertise necessary to support effective planning ▪ The need for technical support, training, strengthening scientific research, and capacity building initiatives ▪ Holding workshops ▪ Using from marketing and business media, magazines, and NGO reviews for complementary desk research and more information for stakeholder negotiations preparedness in LMICs
	Coordination	▪ GHD is more difficult for issues in which there is less interdependence among countries than issues in which countries are directly affected by the actions or inaction of their neighbors ▪ Complex and synergistic policies and political relations at both national and international levels ▪ Policy implications not considering country-specific social and cultural factors ▪ Language barriers to interaction ▪ Lack of coordinated strategies and diplomatic initiatives to address the multinational dimension of the issue	▪ Using Media for advocacy and influence on the policy positions of countries ▪ Advocacy to highlight the importance of developing coordinated strategies
	Advocacy	▪ Dealing with the effects of the globalization of marketing strategies of the food and tobacco industry requires stronger engagement especially with developing countries (For example, the export of unhealthy and processed foods high in added sugars and salts from a developed country to a developing country may increase the prevalence of obesity, without impact on people in the developed country)	▪ Using Media for advocacy and influence on the policy positions of countries ▪ Design an effective consumer campaign that includes the participation of all relevant stakeholders and is suitable for different settings
	Financial Support	▪ Limited and unstable funding for building alliances at the global, regional, and local levels ▪ Low national investments and failure to mobilize sufficient funds at the international level; (a large number of policies are developed without the necessary structure and resources for implementation) ▪ Scarcity of resources and competing infectious disease priorities in LMICs ▪ The costs and consequences of inaction are unclear or not fully understood	▪ Increased public-private partnerships ▪ A strategy for increasing the quality and visibility of information about the global economic impacts of chronic disease, which could create incentives for cross-border collaboration ▪ advocacy efforts to seize opportunities for development agencies and international partners that play different roles in supporting LMICs ▪ Translate existing policies into appropriate action plans to ensure LMICs facing multiple health burdens within their specifi c local conditions and contexts ▪ using low-cost ways to prevent and control NCDs and learning from high-income countries and other LMICs cost-effectiveness models and policies and devising their models ​in LMICs
	Legal Mandates	▪ Inability to develop a coherent plan on financial, policy, and institutional issues	▪ Ensuring implementation through legislation, norms and standards, or investment; application of health-in-all-policies, whole-of-government, whole-of-society, and cross-sectoral approaches in actions against NCDs
	Implementation, Monitoring and Evaluation	▪ Lack of awareness by various sectors about their potential contribution, weak political will, coordination complexity, and inadequate resources ▪ Lack of time from different partners who often have additional responsibilities ▪ Different goals and priorities between partner organizations and competition between organizations for resources ▪ Ad-hoc groups instead of sustainable mechanisms	▪ Gathering the right people around the table ▪ The importance of engaging of high-level stakeholders ▪ Creating synergies between international partners ▪ Cooperation in identifying and participating in strong regional partners

**Table 2:** Challenges of GHD for NCDs at the national level

Themes	Subthemes	Challenges	Solutions
**Governance at the national level**	Engagement	▪ Competition among government sectors ▪ Inadequate engagement of relevant government sectors in formulation and implementation stages	▪ Developing and implementing national NCD plans as a key WHO policy option to strengthen national capacity for NCD prevention and control ▪ Design national campaigns to strengthening partnerships engagement (Like national campaign to reduce salt intake in Thailand’s Bahrain, Kuwait and Qatar; Blood pressure campaign in Iran)
	Prioritization	▪ Complexity of prioritizing and implementing interventions to maximize their impact	▪ Strengthening partnerships both within the health sector (e.g., hospitals, clinics, and ministries/departments of health) and beyond the health sector (e.g., civil society, academia, media, and the private sector).
	Financing	▪ Insufficient funds (no budgetary allocation for NCD interventions, with most interventions being implemented within the health sector budget)	▪ More efficient use of existing resources and development of innovative funding mechanisms instead of creating a new global fund
	Legal Mandates	▪ Differing viewpoints and limited experience of governments in setting new regulations ▪ Lack of clear guidelines for engagement with other sectors	▪ Strengthening the role of the government in NCD prevention, developing multisectoral public policies and legal frameworks to reduce NCD risk factors, and strengthening health systems to respond to NCDS
**Health Sector**	Ministry of Health	▪ Inadequate access, lack of prevention and health promotion services, and lack of evidence-based interventions and medicines ▪ Power asymmetry, with health ministries and agencies being less powerful within their governments ▪ Lack of an NCD unit in about 50% of the world’s health ministries, and staff lacking key competencies, especially in LMICs ▪ Tendency of the health sectors in all countries to lead in the design and implementation of joint efforts with other sectors after policies are drafted	▪ Improving primacy health care for NCD prevention and treatment of high-risk individuals ▪ Strengthening health systems to address NCDs, including integration of NCD prevention and intervention into primary care; support for low-cost, sustainable prevention programs, including standardized curricula and digital training programs; development of equitable and affordable treatment; advocacy to raise awareness of NCDs through media campaigns
	Nature of NCDs	▪ Multiple diseases encompassing diverse risk factors, treatment regimens, and affected populations	▪ Generating multisectoral evidence
**Civil Society**	Weak Civil Society	▪ NCDs being neglected by most countries, development agencies, and foundations ▪ Most countries, development agencies and foundations are unaware of industry resistance to change ▪ National and international member societies being dominated by medical professionals and not significantly involving people ▪ Diverse social movements with no clear organizational leader	▪ Strengthening national NCD networks as the main driver of social engagement; ▪ greater focus on advertising via television and Internet

**Table 3:** Challenges of GHD for NCDs at the level of industries

Themes	Subthemes	Challenges	Solutions
**Industry**	Conflict of Interest	▪ Conflict of interest of the tobacco industry and tobacco farming	▪ Mapping actors, interests, and structural characteristics of relevant industries and value chains to find whether a collaborative or regulatory approach is more effective
	Lobbying	▪ Lobbying by the tobacco industry	▪ Building coalition and preparing for negotiations
	Multinational Corporations	▪ Strategies by multinational corporations to increase tobacco consumption in LMICs	▪ Strengthening multilateral cooperation and networking especially in LMICs

The ability of governments to develop an effective health and fiscal policy aimed at improving lifestyle factors and preventing NCDs is directly linked to their negotiating capacity and their ability to build a national consensus [25]. The progress on global health has created opportunities for developing GHD for the prevention and control of NCDs. Table 4 provides the opportunities identified from the reviewed literature, which are classified at international, national, and individual levels. One of the most important opportunities at the international level is the support of international organizations such as the UN and WHO of prioritization of NCDs. Also, the great progress of global diplomacy in the Tobacco Control Framework Convention demonstrate the ability of the international community to advance the goals of NCD diplomacy. At the national level, one of these opportunities is the existence of cost-effective measures for NCD control and prevention.

**Table 4:** Opportunities created through GHD toward NCD prevention and control

Level	Opportunities
**International**	▪ Action against NCDs supports other global health and development priorities ▪ The ability to generate political will for tobacco control, which indicates the ability of the international community to cooperate on NCD prevention and control and shows that lessons from tobacco control can and should be applied to other major NCD risk factors ▪ Broad international support for addressing NCDs ▪ Establishing extensive scientific collaboration worldwide and creating a common learning environment ▪ Key impacts of recent GHD on tobacco control, including global mobilization of civil society in support of the Framework Convention on Tobacco Control (FCTC) and the emergence of a large coalition known as the Framework Convention Alliance (FCA) ▪ The unique context provided by FCTC negotiations for examining the role of NGOs in GHD ▪ Success of FCTC negotiations, which suggests that civil society can help facilitate cooperation among countries within the UN system
**National**	▪ Access to affordable, cost-effective, and feasible interventions ▪ Presence of national multisectoral governance and coordination structures or a mechanism to oversee NCD policy engagement beyond the health sector, which has facilitated multisectoral action in NCD policy development in some countries ▪ Potential power of multi-stakeholder collaborations for NCD control ▪ Increasing awareness among leaders of the existing scientific evidence on NCD prevention and control and of internationally accepted best practices in NCD-control planning ▪ Working with international partners in bringing the right players to the table ▪ Governments represent power in diplomacy, while NGOs represent ideas and knowledge ▪ The role of FCA in agenda-setting for tobacco control in many countries. ▪ Strengthening public health authorities’ political position; evidence that interdependence is not the only incentive for state actors to participate in GHD
**Individual**	▪ Civil society organizations pressuring highly influential people

## Discussion

Today’s world is more complex and interconnected than ever before. This requires countries to understand their interdependence and common interests. Without a deep understanding of this issue, governments will not be able to interact properly at the international level. There is also a need for collaboration based on a non-zero-​sum game or win-win strategy. Governments should align their national interests with regional and global interests and work to achieve them through collaboration [26–28]. Diplomacy, especially global health diplomacy (GHD), will be crucial in this regard. Given the complexity and challenging nature of addressing NCDs at the national and international level, this systematic review was conducted to identify the challenges and opportunities in GHD for NCDs. Between 2007 and 2020, 32 articles have been published concerning the challenges and opportunities in GHD for NCDs. It should be noted that, the analysis of this article uses specific information in the reviewed articles and may not be able to gather all the complex discussions which are written in the gray literatures.

The identified challenges were classified into three levels: global (global health governance), national (governance at the state level, health sector, and civil society), and industry. At the international level, insufficient scientific research [29], lack of technical expertise, insufficient information on developing and least developed countries, and potential differences between national and global priorities are some of the challenges that many countries face. For example, In India, solving the problem of childhood obesity is considered a kind of luxury. This creates a possible inconsistency between national and global priorities. This is reflected in the fact that for most Indian, more calories are preferable to fewer calories, which is the opposite of one set of recommendations [30] .

At the international level, there is power asymmetry in international negotiations. This is exacerbated in the context of GHD for NCDs, as health ministries and agencies are often less powerful within their own governments. Coalition building and preparation for negotiations are two strategies that have been proposed to overcome these challenges [12]. Moreover, GHD is more difficult for issues in which there is less interdependence among countries than issues such as NCDs in which countries are directly affected by the actions or inaction of their neighbors [13]. There are also complex and synergistic policies and political relations at both national and international levels [31]. As the Caribbean Community (CARICOM) has done to put NCDs on the UN agenda, or the European Union has done recently in response to the COVID19 pandemic, regional groups can play a key role in promoting global health by changing political relations and integrating advocacy efforts [32].

At the national level, the prioritization and implementation of interventions to maximize their impact is complex and requires effective partnerships both within the health sector (e.g., hospitals, clinics, and ministries/departments of health) and beyond the health sector (e.g., civil society, academia, media, and the private sector) [33]. For instance, community-Based Cardiovascular Disease Prevention Project in Finland (North Karelia Project) successfully used a multi-sectoral approach to reduce the risk factors associated with cardiovascular disease. This comprehensive intervention included health education and community empowerment, improving health care delivery, prevention efforts in several settings (schools, workplaces), media participation, with greater involvement of civil society and the private sector. Public health policies also played an important role in promoting low-fat options by regulating food labeling, tobacco regulations, and changing agricultural subsidies [34]. In addition to reducing risk factors, these multisectoral collaborations involve a large number of stakeholders at various levels, play a role in the development of multilateral and informal diplomacy, and change attitudes and influence the country’s public policy.

There is also a lack of awareness across sectors regarding their potential contribution. Ineffective health systems and poor economies in LMICs have made it difficult to address NCDs. Many countries lack the necessary technical expertise, resources, research capacity, and data to overcome the challenges posed by NCDs [35]. Scarcity of resources and competing infectious disease priorities in LMICs have complicated efforts to prevent and control NCD in these countries. Moreover, sanctions, including financial, travel and trade restrictions, are important barriers to these interactions [33, 36].

National investments are often low, and sufficient funds are not mobilized at the international level. Usually, a large number of policies are developed without the necessary structure and resources for implementation [36]. Resource gaps in many countries can be addressed through innovative funding mechanisms and more effective use of existing resources. Many NCD interventions are profitable, including alcohol and tobacco taxes that simultaneously reduce NCD risk and help reduce inequalities [36]. One of the challenges in tackling NCDs is the complexity of these diseases as they have a wide range of risk factors from global to local levels. In addition, not all NCDs are preventable. Due to these complexities, it is difficult to specify the goals and required funds for the prevention and control of NCDs. The global response to these diseases must focus on generating multisectoral evidence on the transnational factors that contribute to the rise in NCDs and the policies proposed for controlling them [19].

Another problem facing health systems is that 50 % of the world’s health ministries lack an NCD unit, especially in LMICs, and in those ministries that have an NCD unit, the staff often lack the necessary competencies [33]. Weak health systems, inadequate access, lack of prevention and health promotion services, and lack of evidence-based interventions and medicines are other challenges that hinder NCD prevention and control [35]. ALSO, Many developing countries have different major organizational structures with functions related to non-communicable diseases. Cambodia and the Philippines, Mongolia for example, have an independent national health promotion center. Many countries, including Malaysia, Mongolia, and the Philippines, have established health promotion committees to provide population-based primary prevention services for noncommunicable diseases. However, the relationship of these newly proposed structures to the existing organizational structures for health promotion or NCDs units in the Ministry of Health is not clearly defined. High potential overlap has been reported in the functions and responsibilities of these structures [37]. If the common goals stated in specific NCDs policies and programs are to be pursued effectively and efficiently, clarify the roles and responsibilities of each institutional structure involved to coordinate non-communicable disease functions, activities and resources across multiple organizational structures; Distinctive needs throughout the Ministry of Health.

On the other hand, power asymmetry and the fact that health ministries and agencies are often less powerful within their governments reduces the impact of political and diplomatic efforts, especially in intersectoral diplomacy. In some countries, the presence of national multisectoral governance and coordination structures or a mechanism to oversee NCD policy engagement beyond the health sector has facilitated multisectoral action in NCD policy development [38]; therefore, an effective solution for addressing this challenge is to develop multisectoral governance structures to tackle NCDs and their risk factors. To engage in the discourse of GHD, NGO diplomats face two immediate challenges: to convey the interests of the general public while contributing to inter-state negotiations in a system of governance that is state-centered and under pressure from private interests or the self-interest of government organizations [39]. According to Bond, one of the main goals of GHD is to “ensure dialogue with affected communities and be more intentional in engaging citizens and groups in defining needs and goals” [40].

Another major challenge is that there are diverse social movements with no clear organizational leader [19]. Moreover, national and international member societies are dominated by medical professionals and do not significantly involve people [12]. Civil society and NGOs are a key part of GHD for NCD prevention and control, and their strong presence on the global stage is indicative of their significance [41]. Another key factor in GHD is the presence of advocacy groups and activists in the area of NCDs. One of the most important lessons drawn from previous successes in GHD is that mobilization of civil society is key to ensuring a strong collective response. This has been recognized as a central element in FCTC negotiations [42]. The rise of the large coalition of NCD Alliance (NCDA) can help prioritize NCDs at every level of health diplomacy. Health diplomacy means building consensus and collaboration between governments and NGOs that operate at the community level and have the greatest access to people in need [43].

At the industry level, one of the most important challenges is the conflict of interest of various industries on health and NCD risk factors, including tobacco and alcohol industries. Conflict of interest can hinder or halt policy development and engagement of various sectors. One of the negative impacts of conflicting interests is the industry interference in the development and implementation of policies. Specifically, the interference of the tobacco industry with the policy process has been evident in almost all LMICs [44]. Multinational tobacco companies will continue to increase tobacco use in LMICs. However, there has been a lack of emphasis on NCD control by various sectors at national and global levels on issues such as government and NGO funding for research and prevention as well as development of coordinated strategies and diplomatic initiatives to address the multinational dimensions of the problem [19].

Countries must adopt a strong advocacy and communications strategy on multisectoral action for NCD prevention to raise awareness of NCDs in different sectors and address issues of conflict. They must implement strategies for addressing industry interference that hinders NCD prevention measures [36].

Recent efforts by the UN, WHO, and world authorities have created opportunities for tackling NCDs. One example of these efforts is WHO guidelines and recommendations. These recommendations are non-binding, but represent the official policy of this organization and define certain norms and standards [29]. Human rights approaches, equity-based approaches (non-discrimination, gender equality, participation), multisectoral and multi-stakeholder action, health-in-all-policies, whole-of-government and whole-of-society approaches, appropriate management of conflicts of interest, national action supported by international cooperation and solidarity, life-course approach, empowerment of individuals and communities, evidence-based strategies, and universal health coverage (UHC) are some of these recommendations. Access to affordable, cost-effective, and feasible interventions against NCDs support other health and development priorities [42].

GHD includes new forms of collective action and negotiation on new rules and norms for tackling global health challenges. The development of national policies and FCTC negotiations have clearly interacted. Thailand, Brazil, and the European Union followed Canada in adopting large graphic health warnings [44]. Until recently, GHD for NCDs was primarily WHO-centric. The UN High Level Meeting was an excellent opportunity for accomplishing the challenging task of elevating NCDs beyond the traditional health forum. An important consideration in decisions to engage in GHD on a given issue is the impact of the media on the political agenda. The media plays a more prominent role in putting issues on the foreign policy agenda than the domestic policy agenda [45].

## Conclusion

Today, health has become an integral part of foreign policy and global agendas on security, global economy, and social justice. Reinforcing health as a social value and a human right, supporting the United Nations millennium development goals, advocating for access to medicines and primary health care, and calling for high income countries to invest in a broad range of global health initiatives are some of the key elements of these agendas [46]. Therefore, advocacy, building coalition with civil society, use of negotiation and diplomacy by health policy makers to engage with trade policy makers regarding the health impacts of international trade agreements, and management of industry conflicts are crucial for prevention and control of NCDs. There is a need for a more coordinated approach at the international level and a greater involvement by health systems and policy makers worldwide. On the other hand, developing a strategic document for public diplomacy, promoting national and transnational capacity building and cooperation, laying the groundwork for expansion of private-sector activities, and leveraging foreign policy to promote national health and welfare can play a key role in the prevention and control of NCDs at the national level. It is also necessary to build a national consensus, align interests with the country’s diplomacy, and monitor the global arena to take advantage of potential opportunities. Also, the use of training and research tools for translating foreign policy into action for NCD prevention and control can pave the way for reducing the burden of these diseases.

## Supplementary Information

**Additional file 1.**

## Data Availability

Data relevant to the study are included in the article or uploaded as Additional files. The datasets used and analysed (include template data collection forms; data extracted from) available from the corresponding author on reasonable request.

## Abbreviations

NCDs: Noncommunicable diseases
GHD: Global health diplomacy
UN: United Nations
WHO: World Health Organization
LMIC: Low and middle-income countries
NGOs: Non-governmental organizations
APEC: Asia-Pacific Economic Cooperation
PAHO: Pan American Health Organization
CARICOM: The Caribbean Community
FCTC: Framework Convention on Tobacco Control
NCDA: Noncommunicable diseases alliance
SDGs: Sustainable development goals
