# Supplementary material for: Opportunities and challenges of global health diplomacy for prevention and control of noncommunicable diseases: a systematic review
Source: BMC Health Serv Res. 2021 Nov 4;21:1193. doi: 10.1186/s12913-021-07240-3 (PMC8567539; doi:10.1186/s12913-021-07240-3)
Supplement: Supplementary file 1 — Additional file 1. [file 12913_2021_7240_MOESM1_ESM.docx]

**supplementary file 1**

| **PICO** | |
| --- | --- |
| **Participants**  **And**  **problem** | **without restriction to countries, population or participants may belong all context**  **NCDs and their risk factor** |
| **interventions** | **global health diplomacy** |
| **Comparison** | **Only reviews comparing GHD interventions with usual situation was included for identifying Opportunities and challenges** |
| **Outcomes** | **The study classified findings based on opportunities and challenges of global health diplomacy for prevention and control of NCDs in different levels** |
